# Supplementary figures and images for: Palmitoylation of the envelope membrane proteins GP5 and M of porcine reproductive and respiratory syndrome virus is essential for virus growth
Source: PLoS Pathog. 2021 Apr 23;17(4):e1009554. doi: 10.1371/journal.ppat.1009554 (PMC8099100; doi:10.1371/journal.ppat.1009554)

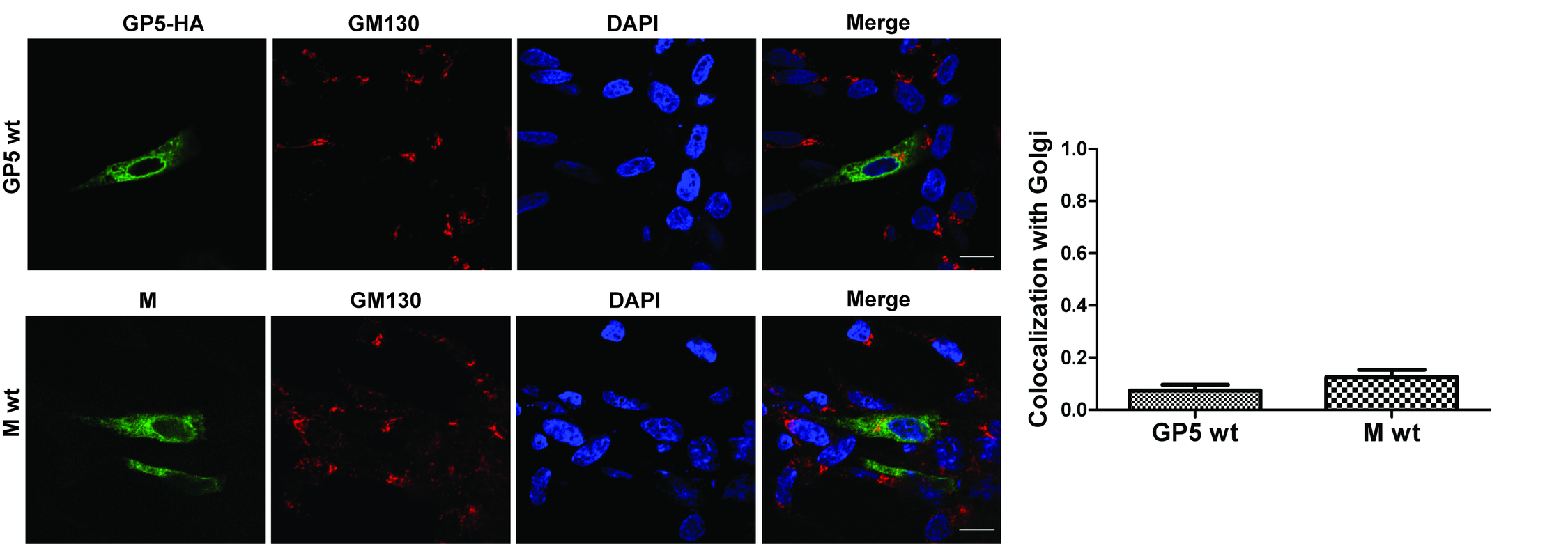

Supplement: S1 Fig — BHK-21 cells were transfected with GP5-HA wt (upper panel) or M-His wt (lower panel). GP5 was stained with monoclonal antibodies against the HA-tag, M with monoclonal anti-M antibodies in both cases followed by secondary anti-mice antibodies coupled to Alexa Fluor-488. The cis-Golgi was stained with polyclonal antibodies against GM130 followed by secondary anti-rabbit antibodies coupled to Alexa Fluor-568 and the nuclei by DAPI. Right panel: Co-localization of M or GP5 with GM 130 from at least 40 cells was quantified with the Pearson’s correlation coefficient method using the JACoP plugin of the ImageJ software. (TIF) [file ppat.1009554.s001.tif]

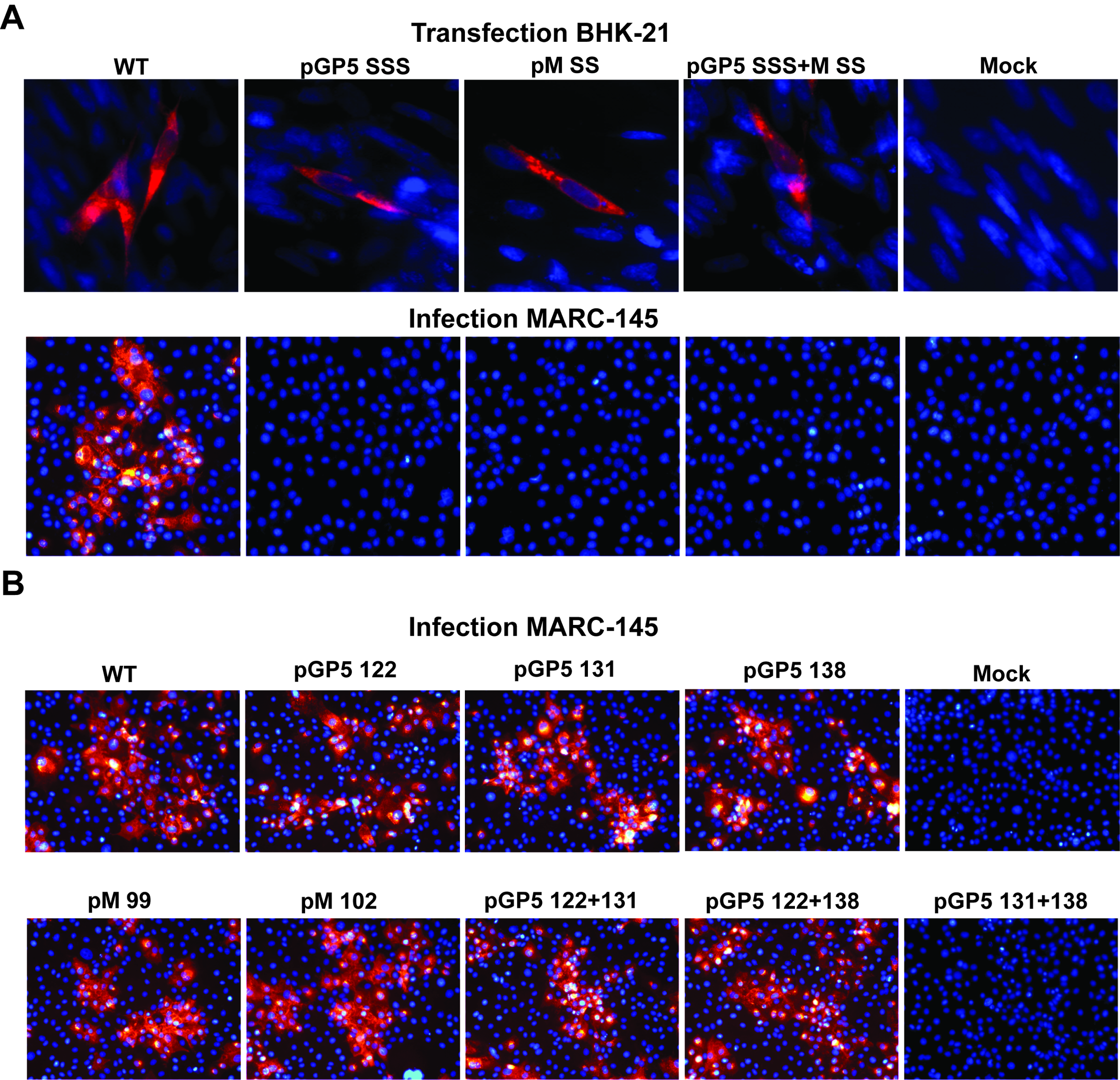

Supplement: S2 Fig — (A) Analysis of mutants where all cysteines in GP5 and M are exchanged. BHK cells were transfected with the viral genome of the XH-GD strain (wt) or with the genomes of mutants where the three cysteine in GP5 (pGP5 SSS) or the two cysteines in M (pM SS) or cysteines in both proteins (pGP5 SSS+M SS) were exchanged to serine. After 48 hours cell supernatants were removed and used to infect MARC-145 cells, which were processed for immunofluorescence 48 hours later. Transfected and infected cells were permeabilized and stained with anti-GP5 monoclonal antibody and Alexa-568 anti-mouse secondary antibody and the nuclei with DAPI. (B) Analysis of mutants where one or two cysteines in GP5 and one in M were exchanged. One cysteine in M (pM 99, pM 102, one cysteine in GP5 (pGP5 122, GP5 131, GP5 138) or two cysteines in GP5 (GP5 122+131, GP5 122+138, GP5 131+138) were exchanged to serine. MARC-145 cells were infected with the supernatant from transfected BHK-21 cells and 48 h later stained with anti-GP5 monoclonal antibody and Alexa-568 anti-mouse secondary antibody and the nuclei with DAPI. Note that only the double mutant GP5 C131+138, that is not palmitoylated in the closely related VR-2332 strain (Fig 2H) could not be generated. (TIF) [file ppat.1009554.s002.tif]

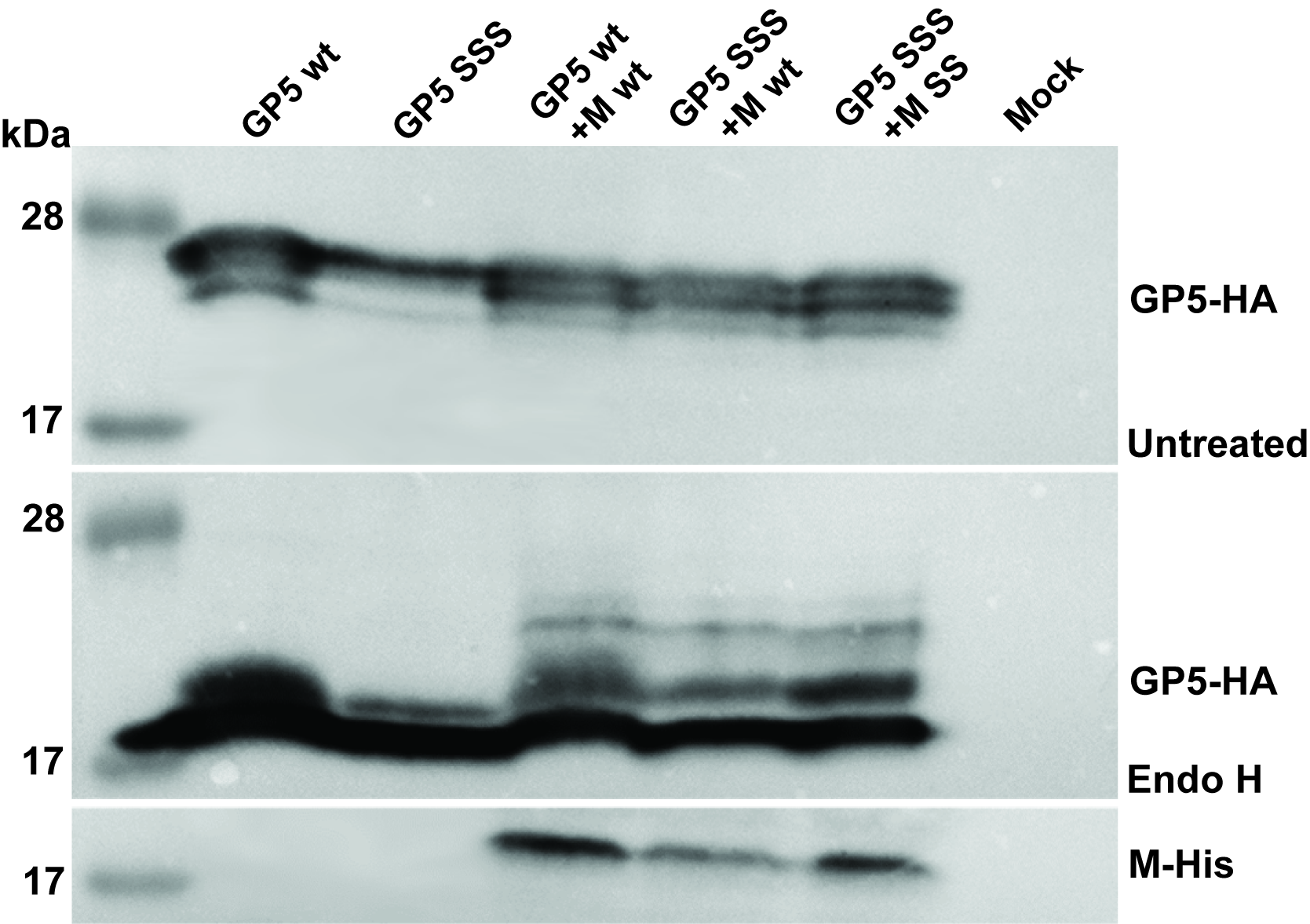

Supplement: S3 Fig — BHK-21 cells were transfected with plasmids encoding GP5-HA wt, non-acylated GP5-HA SSS, M-His wt or non-acylated M-His SS from the VR 2332 strain. 20 hours after transfection cells were lysed and digested with Endo-H or left untreated as indicated. Samples were subjected to SDS-PAGE and western-blotting with anti-HA or anti-His antibodies. (TIF) [file ppat.1009554.s003.tif]

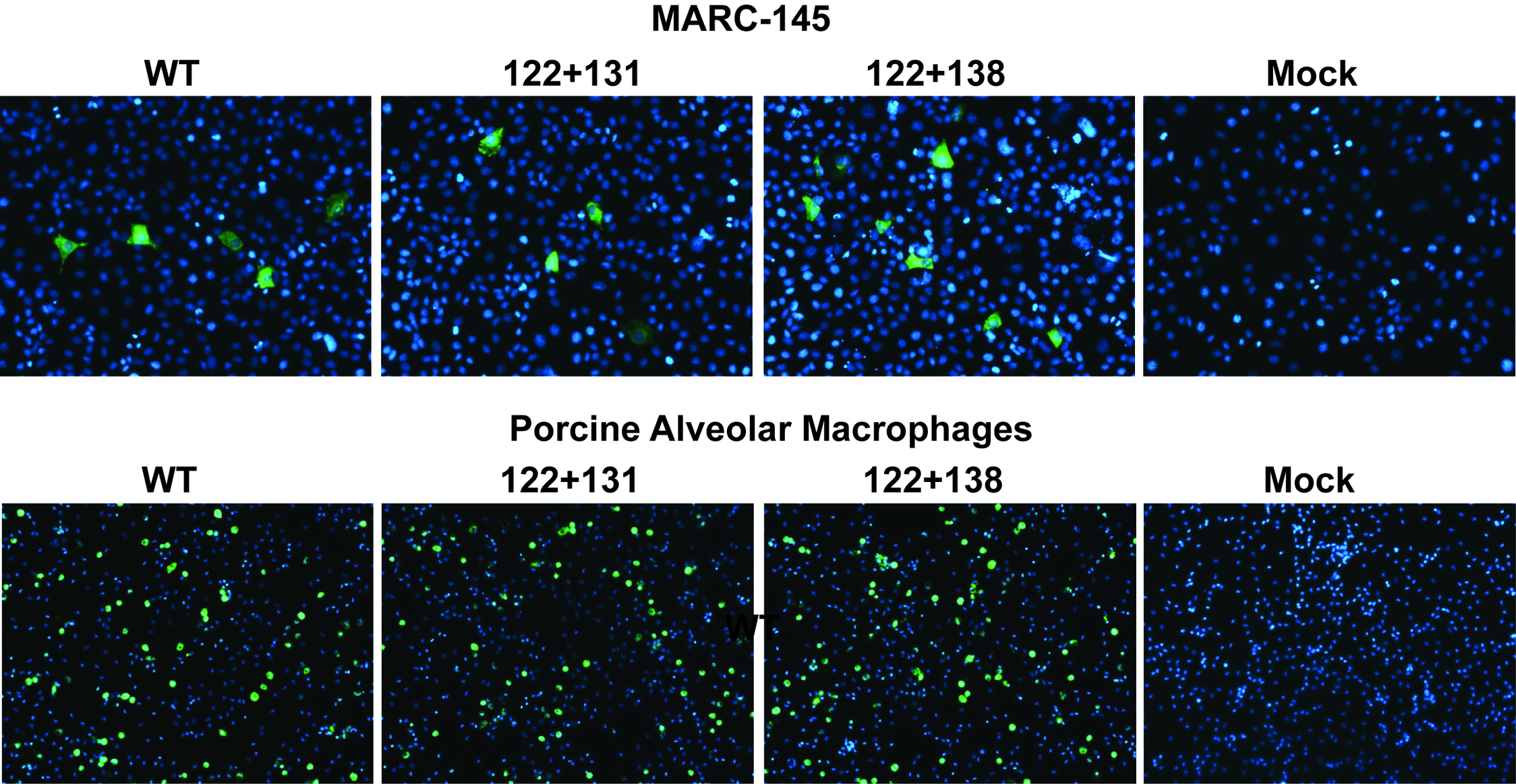

Supplement: S4 Fig — 1x10^5^ Marc cells or 4x10^6^ PAMs were infected with 1x10^5^ virus particles as determined by qRT-PCR. 10 hours after infection cells were fixed, and stained with mouse anti-N antibody followed by anti-mouse IgG antibody coupled to Alexa 568. Pictures were recorded using a ZEISS Axio Vert. A1 inverse epifluorescence microscope. (TIF) [file ppat.1009554.s004.tif]
