# Supplementary material for: Palmitoylation of the envelope membrane proteins GP5 and M of porcine reproductive and respiratory syndrome virus is essential for virus growth
Source: PLoS Pathog. 2021 Apr 23;17(4):e1009554. doi: 10.1371/journal.ppat.1009554 (PMC8099100; doi:10.1371/journal.ppat.1009554)
Supplement: Attachment — Submitted filename: Response to reviewers.docx [file ppat.1009554.s005.docx]

**Reviewer #1:**

**1**. Fig. 1 A: PRRSV-1 strain Lelystad and PRRSV-2 strains VR-2322 and XH-GD serve as prototype references strains. This feels like a very surface level analysis. Given that considerable genetic variation exists between different PRRSV isolates, the authors could strengthen this analysis with a broader panel of sequenced PRRSV isolates. Furthermore, are these conserved cysteines found in other arteriviruses (EAV, LDV, SHFV, etc.). Is this conservation unique to GP5/M of all arteriviruses? Are there exceptions? This information should be provided up front, rather than waiting until the discussion section.

We performed an amino acid sequence comparison of ~9000 GP5 and ~300 M genes present in the database. A percent identity plot for each amino acid position of M and GP5 for PRSSV-1 and PRRSV-2 strains is now added to figure 1. The results show that every acylated cysteine is 100% conserved. Furthermore, we also included the previous figure 8 (sequence comparison between different Arteriviruses) in figure 1.

**2.** RE: “Palmitoylation of M and GP5 was also detected during replication of both viruses in isolated porcine alveolar macrophages, the target cells of PRRSV in pigs (not shown).” PAMs are a more relevant model system than monkey kidney cells (MARC-145), yet that data in PAMs is “not shown.” Either this data should be presented, or this sentence should be removed.

Re: We included the results in the revised manuscript as Fig. 2B.

**3.** “…in contrast to the diffuse bands of GP5 in virus infected cells.” In a later figure, GP5 shows up as a double-band under similar reducing conditions. Is this an exposure issue? **Are these “diffuse bands” being overexposed in the western blot for 1B compared to 1C? The authors should carefully compare viral infections (which express both GP5/M) to transfections with both GP5/M genes, as well as each gene alone.**

Fig. 1B (now Fig. 2A) is indeed overexposed to make the M protein present on the same membrane visible. Nevertheless, we mostly observed a double band for GP5 in virus-infected cells, more pronounced in MARC cells compared to PAMs (see Fig. 6), and a single band in transfected cells (e.g. Fig. 3).

We performed the requested experiment as described below, point 4.

**4.** RE: “The results suggest that GP5 and M remain in the ER if expressed alone, similar to what has been reported for…” **To make this statement, the authors should repeat the experiment, but with co-transfection of both GP5/M.** I think the authors are making too great of a leap here so early in the paper, and this whole paragraph seems out of place. The difference in banding patterns could be due to differences between cell types used, for instance, as N-glycosylation is known to be cell type dependent. However, they then go on to do IF to test their assumptions, but for some reason they switch from MARC-145 (infection) to 293Ts (transfection), and then to making assumptions on GP5/M co-localization with cellular markers in a completely different tissue/species cell line (transfected BHKs) (Supplementary Fig. 1). There should be consistency between the cell types used in these experiments. Further, the authors are measuring co-localization with a Golgi marker and making assumptions about ER localization? **The authors could easily test this assumption by repeating the experiment, but with an ER marker.**

We added another reference for the ER localization of GP5 and M:

“The results suggest that GP5 and M remain in the ER if expressed alone, **confirming previous results** {Wissink, 2005 #25} and as also reported for M and GP5 of Equine Arteritis Virus (EAV) (28).”

Nevertheless, we now compared single and co-expression of GP5 and M, both wild-type and non-acylated proteins. If expressed alone GP5 remains completely Endo-H sensitive, regardless of whether it is acylated or not. Upon co-expression of GP5 with M a small fraction of the total GP5 population becomes Endo-H resistant, independent of whether GP5 or M are acylated. The results are now shown as supplementary figure 3.

Regarding the colocalization experiment: Our intention for this experiment was to get an idea how much of GP5 and M is transported to the Golgi, where acylation of other viral glycoproteins occurs.

To make this clearer we added the following sentence to the text:

**“Acylation of other viral glycoproteins, such as hemagglutinin of Influenza virus occurs in an early Golgi-region by enzymes located in the same compartment {Gadalla, 2020 #244;Veit, 1993 #239}.** To test how much of GP5 and M is transported to the Golgi, we analysed co-localization of GP5-HA and M-His of the VR-2332 strain with the cis-Golgi-marker GM 130 in transfected BHK cells by confocal microscopy. “

Regarding the cell types used: MARC cells are the only cell line that supports growth of PRRSV, but they are difficult to transfect. Hence, they were used for infection experiments. Now we also added some infection experiments with primary porcine alveolar macrophages (PAM) cells, primary cells that need to be isolated from pigs. 293T cells are easy to transfect and hence they were used for the acyl-Rac assay, which requires high protein expression. BHK cells were used for immunofluorescence, since another arterivirus (EAV) can be grown in this cell line. They also exhibit a small nucleus and a large cytosol and hence are suitable for visualization of ER and Golgi, whereas the cytosolic part and hence the ER and Golgi are rather small in MARC cells

**5.** Fig 2. I appreciate the addition of Flotillin 2 as an internal assay control. **This should be “flotillin” not “flotilin" though. It is correct in the text, but incorrect in the figure**. Where is the flotillin blot for 2G? **Aesthetically speaking, the images should all be cropped similarly**. Sometimes the ladder is shown, sometimes it isn’t. Sometimes the 36 kDa band on the gel is shown, sometimes it isn’t. Most blots have a mock, but panel F doesn’t. The consistency in the figure is lacking.

We corrected the spelling of flotillin in the figure. Figure 2F (now 3F) has no mock sample, but evidence for the specificity of the antibody is provided in all the other blots.

**6.** Fig. 2. When each cysteine is replaced, there is a qualitative difference in the amount of GP5 pulled down. This is a really interesting finding, especially given that some cysteines seem to be more important than others in terms of their relative palmitoylation state**.** I’m really curious why the same wasn’t done for M? Similar experiments for GP5 and M are carried on throughout the paper, but for some reason, not here.

I am not sure what the objection is. I do not see a difference in the amount of GP5 wt and non-acylated GP5 in the western-blot (now Fig. 3B and D). There is more non-acylated M present compared to wt M (Fig 3A and C), which, however, does not impair our conclusion that all two acylation sites need to be removed to get a completely non-acylated protein.

The palmitoylation sites in M were analyzed in Fig. 4D. More protein is produced in virus-infected cells, which facilitates the quantification of the acylation levels with the acyl-Rac assay.

**7.** Fig 2 legend. I’m curious about how the statistics were performed. If each independent experiment was normalized to GP5 wt set at 100%, and then that data was combined, your wt would be 100% with no statistical deviation (as presented in the graph). Then, wouldn’t any statistical comparison you make between your mutants and wt here show a significant difference (given the lack of error associated with the wt values)? It seems like the combined, non-normalized data, from three experiments (ratio of +HA to TE; which would also include the variation in the GP5 wt between experiments) would be the dataset that the statistics should be tested on. Not the normalized data. The authors need to be more specific in how their stats were analyzed in the legend and correct if need be.

We now performed the statistics as suggested to show the variation in GP5 wt between experiments. An Anova test performed on this dataset revealed that the acylation levels of GP5 with the two peripheral cysteines removed is no longer statistically significant different from wt GP5. The old figure 2 was replaced by the new figure, which is now fig 3. However, this change in the significance level does not change our conclusion from this experiment “that all three cysteines in GP5 are acylated, but especially the central cysteine is most important for efficient palmitoylation.”

We also revised the normalization for Fig. 4d (acylation sites of M).

**8.** It would be nice to see more consistency throughout the paper. Figure 1 has Lelystad (PRRSV-1) and VR2322 and XH-GD (PRRSV-2). XH-GD is dropped in figure 2, but then viral infections were done with XH-GD through the rest of the paper. For consistency purposes, it would have been nice to also see data for XH-GD in Fig 2 so analogies could be drawn with the same mutants used in transfection/infection experiments.

Sequences encompassing the acylation sites of M are identical between PRRSV-2 strains VR 2322 and XH-GD, and GP5 proteins differ in just one amino acid, a conservative exchange from Ala to Ser (Fig. 1B). Therefore, it seems highly likely that the acylation level of GP5 and also of M is the same in the VR 2322 and XH-GD strains, since it is even similar between the PRRSV-2 strain VR2322 and the PRRSV-1 strain Lelystad, which exhibit large amino acid differences in the acylation region.

**9. Fig. 3 results section:**
a. In the text, viral cDNA was transfected into 293T cells, but in the legend and figure, it states that BHKs were transfected. So, which cell line was it?

Sorry, that was a mistake in the text that is now corrected. BHK cells were used for transfection.

b. It is unclear why this transfection step was necessary. If MARC-145 cells “support virus replication,” why then couldn’t all of this been done in those cells? Could the authors briefly provide more experimental details here?

We added the following information to the text:

“Full-length cDNA was transfected into BHK cells, **which lack the receptors for cell entry of PRRSV but can release infectious virus particles**, and 48 hours after transfection the cell culture supernatant was used to infect MARC-145 cells, **which support virus replication, but are difficult to transfect.”**

c. “…five further attempts to rescue infectious mutant virus also failed.” It is unclear what the parameters were here. Did you do the same thing, five different times, or did you troubleshoot it differently? This sentence alone does not provide much help to the reader.

We added the following information to this sentence:

“Likewise, five further attempts to rescue infectious mutant virus, **in two of them we harvested the cell culture supernatant** **72 or 96 hours after transfection**, also failed, **but wild-type PRRSV was rescued in each case (data not shown)**.”

**10. Fig. 3:** A few points…
a. It would be nice to see the nuclear staining in all images. Just showing a black box isn’t informative.
b. Is there a reason why GP5 staining, but not M staining, is shown? It would be important to show that mutagenesis did not disrupt the ability of these viruses to express both of these proteins.
c. “…we could not detect viral genomes for any of the GP5 and M mutants in the supernatants of infected cells, whereas ~107 genome copies of wild-type virus were present.” I feel that this is the most important point here for Fig 3. There’s no point to show IF infection of MARC-145 cells if no virus particles were produced in the first place. Thus, showing infection via immunofluorescence in MARC-145 cells seems meaningless. Further, for the qPCR data, for all viruses in 3B, are similar genome copy numbers detected in the cell supernatant? This would be an interesting addition to this figure.

A: We added nuclear staining to all the images.

B: We used antibodies against GP5 to determine whether cells are infected or not. In figure 2 (now figure 3) we already showed that removal of the acylation sites from M does not prevent synthesis or stability of the protein.

C: We agree that the figure does not provide a lot of information and therefore we shifted it to the supplementals, now supplementary fig 2.

We characterized virus growth for all mutants in figure 4 and for two GP5 mutants we also determined their specific infectivity, genome copy numbers divided by the TCID 50 titer, see figure 9.

**11. Fig. 3A.** For the GP5/M palmitoylation mutants, where no viral genomes were detected in the supernatant: The authors should explore the fate of virions in the producer cells. Do viral genomes still accumulate in the cell? Can you detect intracellular viral particles? Do these proteins get stuck in different organelles, unlike the wild-type proteins which would be expected to traffic normally? More mechanistic underpinnings here would heighten the impact of this work.

Since only a few cells are transfected (supplementary figure 2) it is difficult to follow trafficking of GP5 and M and probably impossible to detect virus particles by electron microscopy.

However, we now performed qPCR analysis that revealed that viral expression vectors containing the mutations in GP5 and M are able to replicate the viral genome, but only PRRSV wt is able to release viral genomes into the supernatant (see Fig. 5).

**12.** **Fig. 4.** I appreciate seeing the growth kinetics for the wild-type and mutant viruses. As an important control, are GP5 and M in the mutants being incorporated into virus particles to similar levels as the wild-type proteins?

In Fig. 8 we show that GP5 C122S+C131S and GP5 C122S+C138S are incorporated into virus particles, at most at a slightly reduced level, but the data are not statistically different from each other.

Regarding incorporation of M we added the following sentence to the text:

“Since at least for EAV it was demonstrated that only GP5/M heterodimers are incorporated into virions we assume that the level of M in virus particles is also not affected by the mutations in GP5 {de Vries, 1995 #44}.”

**13.** **Fig. 4.** The authors need to address the variability between the replicate experiments. I’m very surprised (and nervous) to see that sometimes there are several logs difference noted between wt and mutant (let’s look at 4B wt vs purple line @ 24h), yet there is no statistical difference between those points. This is a massive amount of experimental variation. The authors cannot conclusively say “each mutant virus grows to lower titers when compared to those of wild-type PRRSV” when all mutant viruses (except 122+131 and 122+138) reach the same endpoint titer that is not statistically different than the wild-type virus. This seems to be a misrepresentation of the data, and that selling point is made multiple times throughout the paper. This is one of the most important figures in the paper, yet the data (statistically speaking) doesn’t fit with the authors interpretation.

The high variability between growth experiments is at least partially due to the intrinsic variability of the TCID50 assay.

If we define the endpoint as the time point exhibiting the highest titer (48 hours post infection), there is a statistically significant difference between the GP5 mutants and wild-type virus. We collected samples also at later time points to investigate whether virus growth of mutants is delayed, but not reduced.

We added the following information to the text:

If cysteines were exchanged individually, the reduction in titers was less pronounced and reached between 10- to 100-fold at 48 hours p.i., **the time when the viral titers peaked.**

We also want to point out that all the data with GP5 and M mutants fit very well together. If all acylation sites in GP5 and M are exchanged, we could not rescue virus, if two in GP5 are deleted virus titers are strongly reduced. If one site in GP5 is exchanged the titer reduction is smaller, statistically significant different only at some time points.

**14.** **Fig. 4 methods:** “the authenticity of rescued viruses was confirmed by sequencing, and viruses were further passaged 2-3 times in T175 flask to generate a for virus stock for subsequent experiments.” Was this sequencing performed before, or after virus passaging to generate stocks? How was it performed? Sanger sequencing of a PCR product? Sanger of multiple independently cloned PCR inserts? Next-gen sequencing? Critical details are missing here. I’m curious how stable these mutations were in their virus stocks, and how stringent the authors were in sequencing their stocks is important for data interpretation.

We added the following information to the text:

“The authenticity of rescued viruses was confirmed by **Sanger-sequencing of a PCR-product encompassing the mutation site**, and viruses were further passaged 2-3 times in T175 flask to generate a for virus stock for subsequent experiments. **The presence of the mutation was again verified by Sanger-sequencing.”**

**15. Fig. 4D.** I don’t see the point in just showing the palmitoylation data for M single mutants 99 and 102, in comparison to wild type. If you want to present this level of detail, why then isn’t this being presented for all of the mutant viruses in A and B? I don’t see any rationale for why these two mutants were chosen. Further, the authors state 10 to 1000-fold differences at 48 h, but in the figure, non-significant differences at 12, 24, 72, and 96 hours are noted. This seems like only select information is being highlighted, rather than telling the whole story. The endpoint titer of these mutant viruses are not significantly different than wt, despite the “tendency for lower titers.”

We analyzed the palmitoylation level of the M-mutants not in transfected, but in virus-infected cells because the expression level is higher (see point 6). The palmitoylation level of the Gp5-mutants were already investigated with the expressed protein in Fig. 3.

Regarding the viral titers see our reply to point 13.

**16. Fig. 5.** How have the authors controlled for equal protein loading in these westerns? No loading controls are shown. For the ratios, I would have expected each band in -ME and +ME to have been internally normalized to a loading control (on the same blot), and then those band intensities could be compared -ME to +ME. Why haven’t the authors similarly probed for M protein in these blots, thereby confirming that the 38 kDa band was a GP5-M heterodimer, rather than speculating? Further, and again to touch on consistency, why is the ladder shown in some but cropped out in others?

In this experiment we compare the amount of GP5 monomers with GP5 dimers (now Fig. 6a) or Endo-H resistant with Endo-H sensitive GP5 (now Fig. 6b) within one lane of the blot. Since we do not compare band intensities between different lanes or blotting membranes we think that a loading control is not required.

This is now more clearly stated in the figure legend:

“The optical density of bands in the samples **separated under non-reducing conditions** of this and two other experiments was determined and the percentage of dimer formation was calculated as dimer divided by the sum of all three bands and normalized to PRRSV wt.

We added another explanation plus a reference to the figure legend:

The band visible only under non-reducing conditions and running with the molecular weight of 38kDa is the GP5 M dimer, **since GP5 does not form homodimers** {de Vries, 1995 #44}.

**17.** Connecting Fig. 3 and Fig. 5. No infectious virus is produced in the pGP5 SSS, pM SS, or pGP5 SSS + M SS mutants. In Figure 5, the authors suggest that GP5 dimerizes with M (non-reducing gel, although M blotting is not shown). Is the reason for the lack of secreted pGP5 SSS, pM SS, or pGP5 SSS + M SS viruses because these mutant GP5/M proteins do not dimerize together, thus, budding is impeded? If you pull down GP5, can you detect M and vice versa? More mechanistic details here would add additional strength to this work.

In the revised manuscript we analyzed whether co-expression of GP5 with M resulted in their transport to the Golgi assuming that dimerization is a prerequisite for this transport (see our reply to point 18 and 19). Whereas wild-type or non-acylated GP5 expressed alone remains completely Endo-H resistant, co-expression with wild-type or non-acylated M resulted in the acquisition of Endo-H resistant carbohydrates. This experiment is now shown as supplementary figure 3.

**18.** I would have appreciated Fig. 5 more if the authors would have better described now N-glycan addition and Endo-H protection fit with their interpretation that the “same number of molecules have passed through the medial golgi-complex.” I’m not fully understanding how the data fit with their interpretations. Importantly, “…Endo-H resistant bands were also detected if non-acylated GP5-SSS and M-SSS were co-expressed (data not shown).” Why is this data not shown? The authors lack experimental detail for why these mutants failed to make infectious virus. Does this, along with the co-localization experiment (Fig. 6), suggest it isn’t due to any trafficking issues?

Carbohydrates of glycoproteins become resistant to Endo-H digestion once the enzyme alpha-mannosidase II removes two mannose subunits. Since this enzyme is located in the medial-Golgi the acquisition of Endo-H resistant carbohydrates is used as a maker for transport to the medial-Golgi.

We added the following explanation and a reference to this sentence:

“and thus the same number of molecules have passed through the medial Golgi-complex**, where glycoproteins acquire Endo-H resistance {Stanley, 2011 #278}.”**

**19. Fig. 6.** This figure analyzes localization of GP5/S mutants, for those mutants that could not produce infectious virus (Fig. 3). How does co-localization of GP5 and M in the Golgi allow the authors to conclude that they are indeed dimerized? This is also an overexpression scenario, would is known to cause unintended effects on protein localization.

We added the following sentence to the text.

“Since oligomerisation of glycoproteins is one prerequisite for their export from the ER it is reasonable to assume that complete removal of acylation sites from GP5 and M does not affect their dimerization {Doms, 1993 #88}.”

**20.** Fig. 7. I appreciate that the authors showed incorporation of mutant GP5 proteins in relation to wildtype. How was the blotting normalized for these concentrated viral preparations, especially since the mutant viruses tend to produce lower viral titers than wild-type? Was it by protein input? Careful descriptions are necessary here. Also, why were these particular mutants chosen but not others? In my opinion, this is an important control figure that should be shown for all viruses produced, and it should have been presented several figures back.

We now added the following information to the figure legend:

“Since the viruses grew to different titers the amounts of the virus preparation loaded on the gel was adjusted to give the same band intensity for the GP5 protein.”

We have chosen the two GP5 mutants with two acylation sites exchanged since they exhibit the strongest reduction in virus titers.

**21.** RE: Virus particles with under-palmitoylated GP5 have no significant defect in cell entry. This whole section was very difficult to follow. Two different assays, with two different viruses are being placed into a single table. The table headers aren’t easy to interpret on their own. The infection values are extremely low, and if you are looking at entry, waiting 10 hours to detect N seems very long. I get the point that the authors are trying to make, and it’s an important one, it just doesn’t come off as easily interpretable in the way the data is described and presented. Also, no staining for N is shown (which is integral to interpreting this entry assay), making this experiment difficult to review. If the authors could get a hold of a reporter PRRSV isolate, it would greatly improve their experimental setup here. It could be normalized by genome content, TCID50 could be determined by flow cytometry, and then entry measured by reporter gene expression shortly after infection.

We agree that the table was too complicated and hence we now show the data as graphs (figure 9) As suggested by referee 3 we now also analyzed cell entry into porcine alveolar macrophages, which are more susceptible to virus infection. Nevertheless, we could not detect a significant difference in cell entry between PRRSV wt and the two mutants with two exchanged acylation sites. See our reply to point 3 of referee 3.

We added a figure showing N staining in infected cells as supplementary fig. 4.

**22.** Overall, I don’t see experimental data to explain why palmitoylation mutant viruses behave differently than wild type. To strengthen this paper, I feel that the authors need to delve deeper into that mechanism. To summarize the major figure conclusions:
a. GP5/M are palmitoylated (Fig. 1)
b. Some cysteines are more important for palmitoylation than others (Fig. 2)
c. Removing all cysteines in GP5 or M prevents viral particle release (Fig. 3)
d. Palmitoylation mutants tend to have lower titers (Fig. 4)
e. There is no difference in glycosylation or Endo H protection (Fig. 5)
f. GP5/M mutants co-localize together (Fig. 6).
g. GP5 mutants incorporate similarly to wt into virions (Fig. 7).
h. No defects in entry are noted (Table 1).
None of this data provides mechanistic insights into why GP5/M need to be palmitoylated, other than reduced palmitoylation of GP5/M has a tendency to impact viral titers.

We now quantified the number of viral genomes released into the supernatant of cells transfected with PRRSV infectious clone plasmids, wild-type and mutants lacking the acylation sites in M and GP5. The results (Figure 5) demonstrate that mutant PRRSV clones, which are not able to generate infectious virus also do not cause the release of viral (genome-containing) particles. This experiment adds direct evidence to one of our main conclusions that palmitoylation of GP5 and M is required for assembly and/or budding of virus particles.

**23.** The authors speculate that acylation affects the budding process. Could the authors use an ELISA to prove this point? If less viruses are budding out, this should be quantifiable in a protein ELISA, which can then further be compared to viral genome copies in infected cell supernatant.

We think that the experiment described under point 22 in combination with the virus entry assay (Fig. 9) is considerable evidence that virus budding is disturbed.

24. Figure 8 is not sufficient as a standalone figure, and I would suggest that these alignments be added to the first figure.

We integrated figure 8 in the revised manuscript into figure 1.

**Reviewer #2:** 1. The report suggests a role for palmitate modifications in virus budding and virus particle membrane curvature. These are reasonable suggestions and the role in budding is somewhat supported by excluding other roles for the palmitoylations. **However, there is not much direct evidence for a role for the palmitates in budding**. One suggestion is to perform **EM of the GP5 2-palm-deficient viruses.** Any evidence of misshapen particles, deformed because under-palmitoylation creates aberrant membrane curvatures?

We think EM is not feasible, because Arteriviruses (in contrast to Cornaviruses) lack a distinct morphology due to the lack of large spike proteins and hence budding virions are difficult to identify inside cells.

However, we now quantified the number of viral genomes released into the supernatant of cells transfected with PRRSV infectious clone plasmids, wild-type and mutants lacking the acylation sites in M and GP5. The results (Figure 5) demonstrate that mutant PRRSV clones, which are not able to generate infectious virus also do not cause the release of viral (genome-containing) particles. This experiment adds direct evidence to one of our main conclusions that palmitoylation of GP5 and M is required for assembly and/or budding of virus particles.

**2.** It is hard to accept one of the main conclusions of the paper regarding palmitates in virus budding. “We concluded that palmitoylation of GP5 does not affect incorporation of the GP5/M dimer into virus particles.” This appears to be based on the single palmitoylated GP5 mutant, lacking two palm cys residues. Any analysis of un-palmitoylated GP5 in virus incorporation could not be performed because palm-negative viruses were not obtained. Therefore, how can it be concluded that palmitoylation of GP5 is not required for incorporation into virus particles?

This objection is justified. We changed the sentence in the discussion to the following: “We concluded that GP5 does not need to be fully palmitoylated to be incorporated into virus particles.”

**3.** The PRRSV cDNAs encoding palm deficient GP5 and M can produce viral proteins but do not release infectious particles. **Can the cDNA – transfected cells be complemented with GP5 and M expression vectors, to then produce viruses?** Results from such complementation tests would further confirm findings in this study.

We tried this complementation experiment once, but co-expression of GP5 and M did not rescue release of virus particles. There might be multiple technical reasons for this failure, especially the low number of cells transfected with both the PRRSV expression construct and the GP5 and M plasmids.

**4.** Virus egress via exocytosis is required after budding. **Have roles for palmitates on GP5 and M in forming egress-competent viruses been considered,** or excluded from consideration for some reason?

We added the following sentence to the discussion:

“After budding, particles are transported through the Golgi and finally secreted by the cells. We think that a role of palmitoylation for virus egress is very unlikely, since the fatty acids are now located inside the virus particle and hence are not able to interact with the cellular machinery that controls exocytosis.”

**Reviewer #3:**

1° See under Summary
I have reservations with the technology that was used for demonstrating that the palmitoylation is not important for the entry of the virus. GP5/M is important for the infection of receptor (siglecs)-carrying macrophages. This is not the case for Marc cells. **As a result, a virus titer of a wild type virus stock in macrophages is tenfold higher than in MARC-145 cells. This explains the big difference between the TCID50 and genome copies, as also demonstrated in the present study.** **In order to study the role of palmitoylation of GP5 and/or M it is important to use macrophages that express receptors that interact with GP5/M.**

Furthermore, it is important to note that RT-qPCR does not only detect genomes inside virions but also free genomes. **It would have been better to first eliminate the free genomes by benzonase treatment.**

We performed the cell entry assay with PAMs and indeed found a tenfold higher number of infected macrophages compared to MARC cells if infected with the same number of particles as determined by qRT-PCR. However, the benzonase treatment did not decrease the number of viral genomes compared to our previous experiments meaning that the specific infectivity (TCID 50 divided by genome copy numbers) is still low. Nevertheless, the viral GP5 mutants with two acylation sites deleted revealed basically the same results as wt PRRSV indicating that cell entry is not disturbed.

For clarity and consistency, we now used these new data from three virus preparations in the revised manuscript, which are now shown as a graph (Fig.9)

However, the advice about benzonase treatment was very useful to solve or technical issue with the qRT-PCR method to determine the number of viral genomes released into the supernatant of transfected cells. The results (Figure 5) demonstrate that mutant PRRSV clones, which are not able to generate infectious virus also do not cause the release of viral (genome-containing) particles. This experiment adds direct evidence to one of our main conclusions that palmitoylation of GP5 and M is required for assembly and/or budding of virus particles.

**2°** Macrophages are the real target cell. Therefore, some findings in MARC-145 cells should be confirmed in macrophages such as the demonstration that palmitoylation is not required for dimerization of GP5 and M, for transport to the Golgi apparatus and for incorporation into virus particles.

The revised manuscript now demonstrates palmitoylation of GP5 and M in PAMs infected with PRRSV-2 viruses (Fig. 2B). PAMs were also used to show that removal of acylation sites from M and GP5 does not disturb their trafficking to the Golgi (Figure 6C), similar to what we observed in MARC cells (Figure 6A+B).

**Part III – Minor Issues: Editorial and Data Presentation Modifications**
Please use this section for editorial suggestions as well as relatively minor modifications of existing data that would enhance clarity.

**Reviewer #1: 1.** The manuscript should be updated with line numbers to help the reviewer address specific lines in the paper.

Line numbers were added.

2. Abstract: “no virus could be rescued if all three cysteines present in GP5 or both present in M were replaced in a PRRSV-2 strain, indicated that acylation is essential for virus replication.” I don’t think “replication” is the correct term here. No viral genomes were released for these mutants, but the authors did not show that these mutants had any defect in viral genome replication in the producer cells. Perhaps, viral “egress from the cell” would be more suitable?

Yes, we changed “virus replication” by “virus growth” throughout the text.

3. Fig 1 legend: “Note that GP5 also contain cysteines in the middle of the TMR.” These cysteines are immediately adjacent to the cytoplasmic tail. Is “cysteines in the middle of the TMR” correct here?

This part of the figure legend was deleted since we added a different figure.

4. “…we exchanged them with serine in GP5-HA and M-His in both the VR-2322 and Lelystad viruses.” And here: “…revealed a remaining palmitoylation level of ~20% in the Lelystad and of only ~10% in the VR-2332 virus.” The way these sentences are worded makes it sound like you made these serine mutations in the viruses themselves, rather than expression vectors. Please double-check and correct this wording throughout.

Was changed to:

“we exchanged them with serine in GP5-HA and M-His **from** both the VR-2332 and Lelystad viruses.”

“Quantification of the band densities from this and two other experiments revealed a remaining palmitoylation level of ~20% **in GP5 from** the Lelystad virus and of only ~10% in **GP5 from the** VR-2332 virus (Fig. 2E+G).

5. Fig. 3 legend and throughout: “Alex-568” should be Alexa-568.

Corrected.

6. The manuscript should be thoroughly re-read and corrected for grammar and spelling errors.

**Reviewer #2:** 1. A point about terminology here: The term “replication” often refers to replication of viral genetic material. In this context, then, it is not established that the absence of palmitates eliminates PRRSV “replication”. In fact, replication appears to be demonstrated in the absence of palmitoylated GP5 – M. Palmitates instead are suggested to be needed for particle assembly or budding or perhaps particle egress. The use of the terms “replication” and “replication deficient” might be reconsidered in this manuscript.

We agree that this terminology is misleading. We changed “virus replication” to “virus growth”.

2. Regarding consensus palmitoylation motifs, are there not predictive algorithms for palmitoylation? (intro stating lack of consensus motifs suggests otherwise).

The sentence was misleading.

We changed it to the following: “In contrast to most other protein modifications, no consensus motif that directs palmitoylation has been established and current prediction tools, such as Css palm 4 ( http://csspalm.biocuckoo.org/online.php) are of limited value to precisely predict palmitoylation of viral proteins (40).”

3. Fig. 5; were thiol blockers (NEM or IAA) included during the cell lysis process, to prevent artifactual post-lysis disulfide formations?

For the acyl-Rac assay disulfide-bonds were reduced with Tris (2-carboxyethyl) phosphin (TCEP) and free cysteines were then blocked with methyl methanethiosulfonate (MMTS).

To describe the dimerization experiment we added the following information to the methods section.

To analyze dimerization of GP5, virus-infected MARC-145 cells were lysed **by 0,5 % SDS**, and **quickly boiled** in non-reducing (without mercaptoethanol) or reducing (with mercaptoethanol) SDS-PAGE loading buffer and subjected to SDS-PAGE and Western blot.

We do not think that such a procedure induces massive artificial disulfide-formation.

4. The review process would be more effective if the text included line numbers, so if there is any additional review, a submission with line numbers would help.

Line numbers were added.

**Reviewer #3:** Author summary - last sentence: ‘They might trigger assembly of GP5/M dimers to from…’ change into ‘They might trigger assembly of GP5/M dimers to form…’

Corrected
