# Supplementary material for: Palmitoylation of the envelope membrane proteins GP5 and M of porcine reproductive and respiratory syndrome virus is essential for virus growth
Source: PLoS Pathog. 2021 Apr 23;17(4):e1009554. doi: 10.1371/journal.ppat.1009554 (PMC8099100; doi:10.1371/journal.ppat.1009554)
Supplement: Attachment — Submitted filename: Respone to referees.docx [file ppat.1009554.s006.docx]

**Part II – Major Issues: Key Experiments Required for Acceptance**
Reviewer #2: This reviewer still has a few questions.
1. Fig. 5: Since the vGP5 122 + 138, vM 99 and vM 102 are so prominent in several experiments (shown in Figs 4, 8, 9), why are they not evaluated and included in the Fig. 5 results?

Fig. 5 contains the result with the double mutant pMSS, but not with the single M mutants and also not with vGP5 122 + 138, since the latter produced infectious virus particles, which were characterized in the next paragraphs. Besides wild-type genome we just added another sample (GP5 122+131) to have more than one positive control, i.e. a genomes that are able to generate infectious virus particles.

2. **The new Fig. 5 seems suboptimally placed into the manuscript.** After producing mutant genomic cDNAs, the very first test would seem to be the one whose results are shown in Fig. 5. Then, with the results, the next questions could focus on the characteristics of those few recombinant cys-to-ser viruses that were produced and secreted; i.e., their replication kinetics (Fig 4), virion proteins (Fig 8), entry into target cells (Fig 9) and so on.

That is true. Thus, we have changed the position of Figures 4 and 5 and the associated text in the manuscript.

3. This reviewer still has difficult with summary statements. *The statement at the end of the abstract refers to “clustering of GP5/M dimers at Golgi membranes” and it is not clear how the findings reported in the body of the abstract support this particular statement.*

Other summary statements are hard to discern, for example, lines *47-50 of the author summary; “acylation has no effect on dimerization of GP5 with M, its transport to budding sites, and incorporation into virus particles. We therefore propose that the fatty acids are required for the budding process.”* It would help to clarify how observation of under-palmitoylated GP5 in virus particles lead one to the stated proposal.

We agree that our work does not support this statement, but we wanted to speculate on a possible mechanism in the last sentence. To make this clear we changed the sentence to:

“We **speculate** that multiple fatty acids…”

To be more precise we changed the two sentences of the authors statement to the following:

“The specific infectivity and cell entry of viruses **lacking two acylation sites in Gp5** is, however, not reduced. Likewise, **these viruses** revealed no effect on dimerization of GP5 with M, its transport to budding sites, and incorporation into virus particles. **Since cells transfected with a cDNA expressing non-acylated GP5, or non-acylated M release no virus-like particles into the supernatant** we propose that the fatty acids are required for the budding process.”

**Part III – Minor Issues: Editorial and Data Presentation Modifications**
Please use this section for editorial suggestions as well as relatively minor modifications of existing data that would enhance clarity.
Reviewer #1: 1. I appreciate the addition of Fig. 1A, and it’s fascinating to see this analysis of sequence variation between isolates. However, it’s difficult to discern which dot in the graph each cysteine is referring to given how scrunched together the data points are. To better distinguish the cysteines, could the authors simply mark those data points in a different color?

We now indicate the location of the cysteines as a red dot.

2. The addition of the PAM experiment (Fig. 2B) provides important data for a natural host cell target. However, I’m struggling with the M-protein blot. It doesn’t appear as though a -HA sample was loaded given the lack of spacing between the M-specific bands. Can the authors please comment, or perhaps include a new blot here that is more consistent with the others?

We performed this experiment just once since the M-protein was hard to detect in infected PAM cells. To be able to detect M, infected cells from one T75 flask (instead of two wells from a 6well plate for GP5) had to be processed and subjected to the acyl-Rac procedure. Therefore many acylated proteins (+HA) and also total protein (TE) were loaded onto the gel which spread out to the neighboring-HA lanes. These samples contain less material, since little protein were pulled down by the beads without hydroxylamine catalyzed cleavage of fatty acids. This overloading of the gel with proteins in certain lanes probably led to the distorted band shapes.

3. Lines 138-139: For added clarity, please denote which proteins ORF5 and ORF6 encode.

We added this information to the sentence.

“…1077 ORF5 nucleotide sequences **encoding GP5** from PRRSV-1 strains, 7701 from PRRSV-2 strains, 83 ORF6 nucleotide sequences **encoding M** from PRRSV-1 strains and 290 from PRRSV-2 strains...”

4. Line 176: Suggest replacing “confirming” to “consistent with”

Corrected as suggested.
